# Ultrasonographic and endoscopic guidance in diagnosis of Helicobacter gastritis presenting as a mass lesion in a dog: A case report

**DOI:** 10.3389/fvets.2022.959526

**Published:** 2022-07-29

**Authors:** Thomas Gomes, Cecelia Harmon, Michael Nappier

**Affiliations:** Department of Small Animal Clinical Sciences, Virginia-Maryland College of Veterinary Medicine, Blacksburg, VA, United States

**Keywords:** gastric mass, *Helicobacter*, antibiotics, fundus, lymphocytic-plasmacytic gastritis, ultrasound, endoscopy, dog

## Abstract

This case documents a previously undescribed presentation of *Helicobacter* spp. gastritis. An 8-year-old female spayed golden retriever with chronic vomiting was found to have a cluster of multiple, round, well-defined, hypoechoic foci of varying sizes surrounded by gas within the lumen of the stomach on ultrasonographic examination. Further endoscopic examination revealed multiple raised mass-like lesions in the fundus on endoscopic examination. Histopathological findings were consistent with *Helicobacter* spp. infection. The dog was treated with both amoxicillin 400 mg and clarithromycin 180 mg BID for 21 days and omeprazole 20 mg SID for 34 days. After the treatment, the vomiting and fundic lesions were resolved on ultrasonographic examination. This case represents a novel gross morphologic presentation for *Helicobacter* spp. gastritis that responded to appropriate therapy and highlights how early intervention with advanced imaging can aid in diagnosis and treatment.

## Background

*Helicobacter* spp. have long been known to be associated with canine gastritis, although the exact causes are not described. On pathological survey, *Helicobacter* spp., or non-*Helicobacter pylori Helicobacter* species (NHPHs), have been identified in the cardia, fundus, body, and pylorus of infected dogs (1). Infected canines have been described to be asymptomatic or exhibit clinical signs, such as vomiting and diarrhea (2). Unlike in humans, infection by NHPHs in canines rarely causes ulcerative lesions; more often, they are present in or on the gastric mucosa with no associated pathology, and, in some cases, are associated with lymphoplasmacytic gastritis (1, 3). Previous descriptions of NHPHs gastric infections in dogs have shown no gross lesions or tissue inflammation (2). Histologically, *Helicobacter* spp. infections are described to be associated with lymphoplasmacytic infiltrates, inflammatory infiltrates, and degeneration of the gastric crypts (1, 3). Diagnosis of NHPHs infections has primarily been made through mucosal biopsies of multiple regions of the stomach, which are submitted for tests, such as rapid urease test, histopathology, or PCR (3). Non-invasive techniques for diagnosis have proven to be limited in canines as the number of *Helicobacter* species canines host makes serology challenging, while urea breath and blood tests are most helpful following antimicrobial therapy as they demonstrate eradication of the *Helicobacter* spp. colonies (3). Empiric treatments for NHPHs-associated gastritis in dogs include amoxicillin, metronidazole, and an optional gastroprotectant with a good prognosis (3, 4). This report aims to describe a novel gross mass-like lesion presentation of *Helicobacter* spp. gastritis in a canine on ultrasonographical and endoscopic examination, yet, had typical clinical signs, histopathologic appearance, and response to therapy for NHPHs-associated gastritis.

## Case report

An 8-year-old spayed female Golden Retriever was presented to the Virginia-Maryland College of Veterinary Medicine (VMCVM) community practice for intermittent vomiting for 1-month duration. The owner reported that the dog had similar bouts of vomiting approximately 5 months earlier.

**Figure 1:**
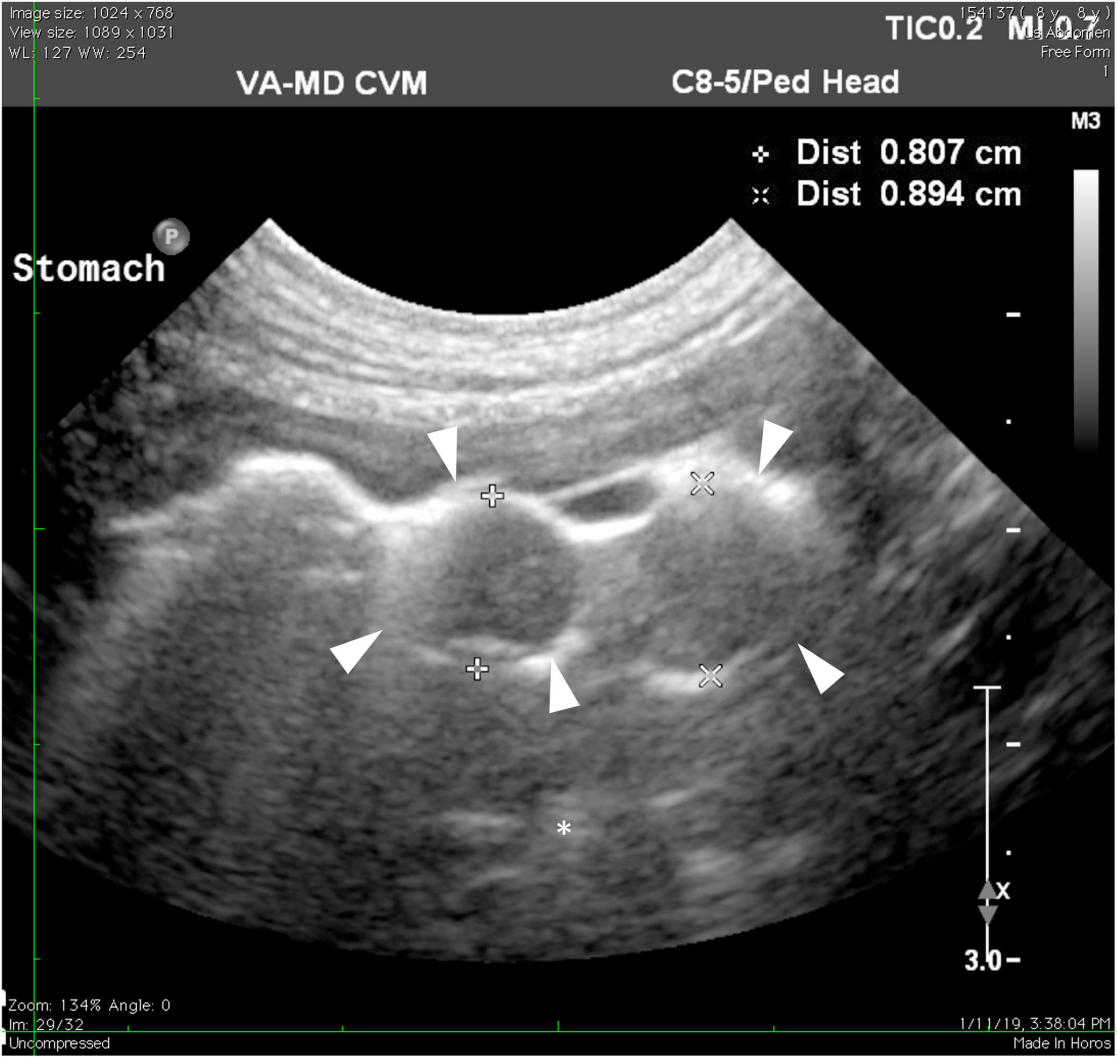
Ultrasonography of the stomach. The hypoechoic masses (arrowheads) protruding toward the lumen of the stomach are less echogenic than stomach contents (asterisk).

On presentation, the patient was in good condition and vital parameters were normal. No abnormalities were found on the physical exam with no pain or other abnormalities on abdominal palpation. Blood was sampled for hematology, biochemistry, thyroid hormone, and serum cortisol to help rule out multiple differentials for chronic vomiting in a dog, such as hypoadrenocorticism, hepatic disease, inflammatory bowel disease, neoplasia, and hyperthyroidism. Hematology revealed a mild microcytosis (58.2 fl) and hypochromia (20.1 pg). Serum biochemistry showed a mild increase in ALT (91 U/L). Serum cortisol (1.01 μg/dl) and thyroid hormone (21.8 nmol/L) were within the reference intervals. Radiographs revealed a focal soft tissue opacity that appeared to fill most of the gastric fundus not identified on lateral views. Other abdominal contents were found to be normal. Transabdominal ultrasonography revealed a cluster of multiple, round, well-defined, hypoechoic foci of varying sizes (~0.7–1.8 cm) surrounded by gas within the lumen toward the fundus of the stomach (Figure 1). These foci were not associated with the wall along the greater curvature. Other abdominal structures were found to be normal.

**Figure 2:**
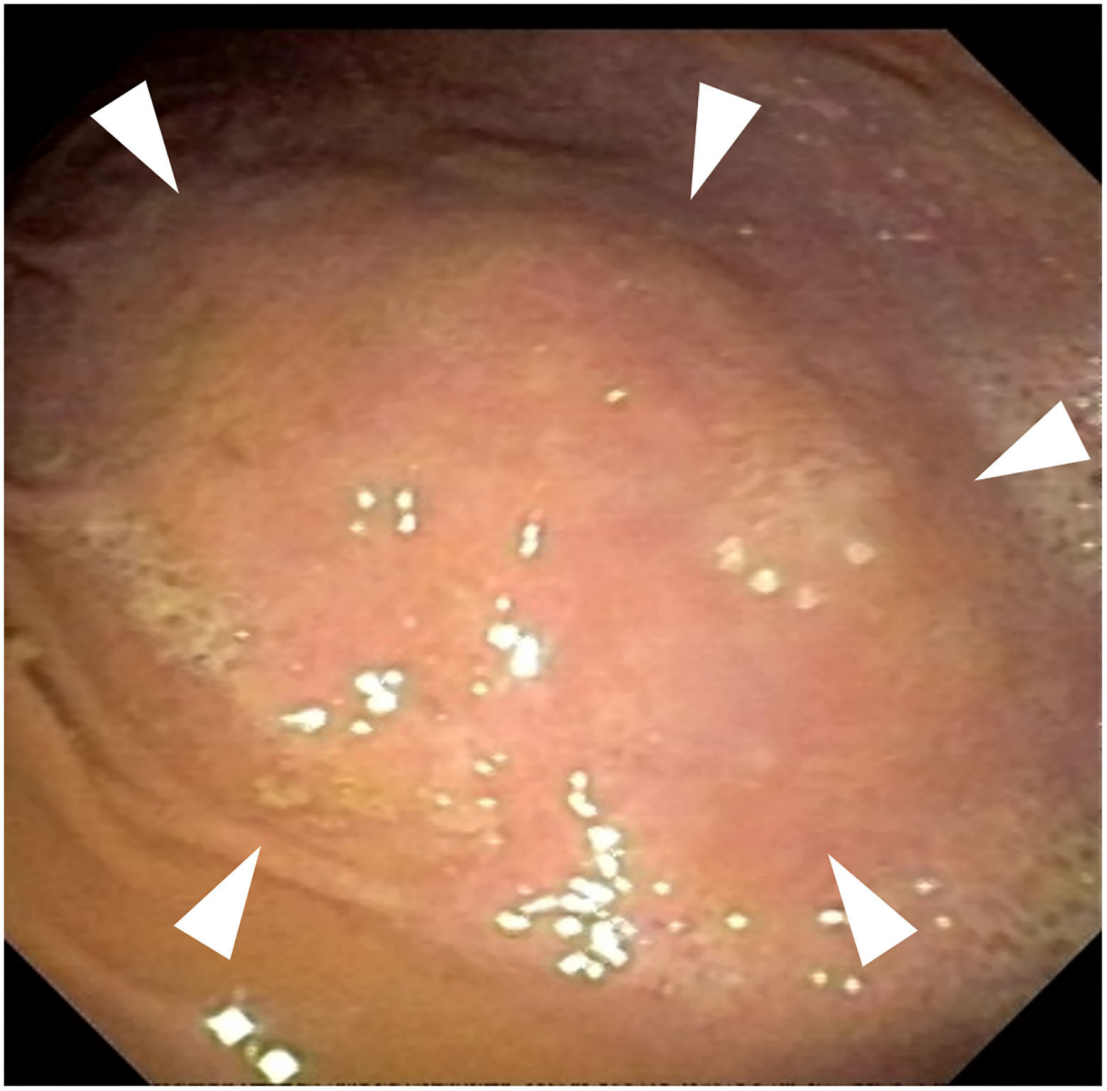
Endoscopy of the fundic region of the stomach. Gross thickened areas (arrowheads) appear to be from the stomach wall and do not involve the gastric lumen.

**Figure 3:**
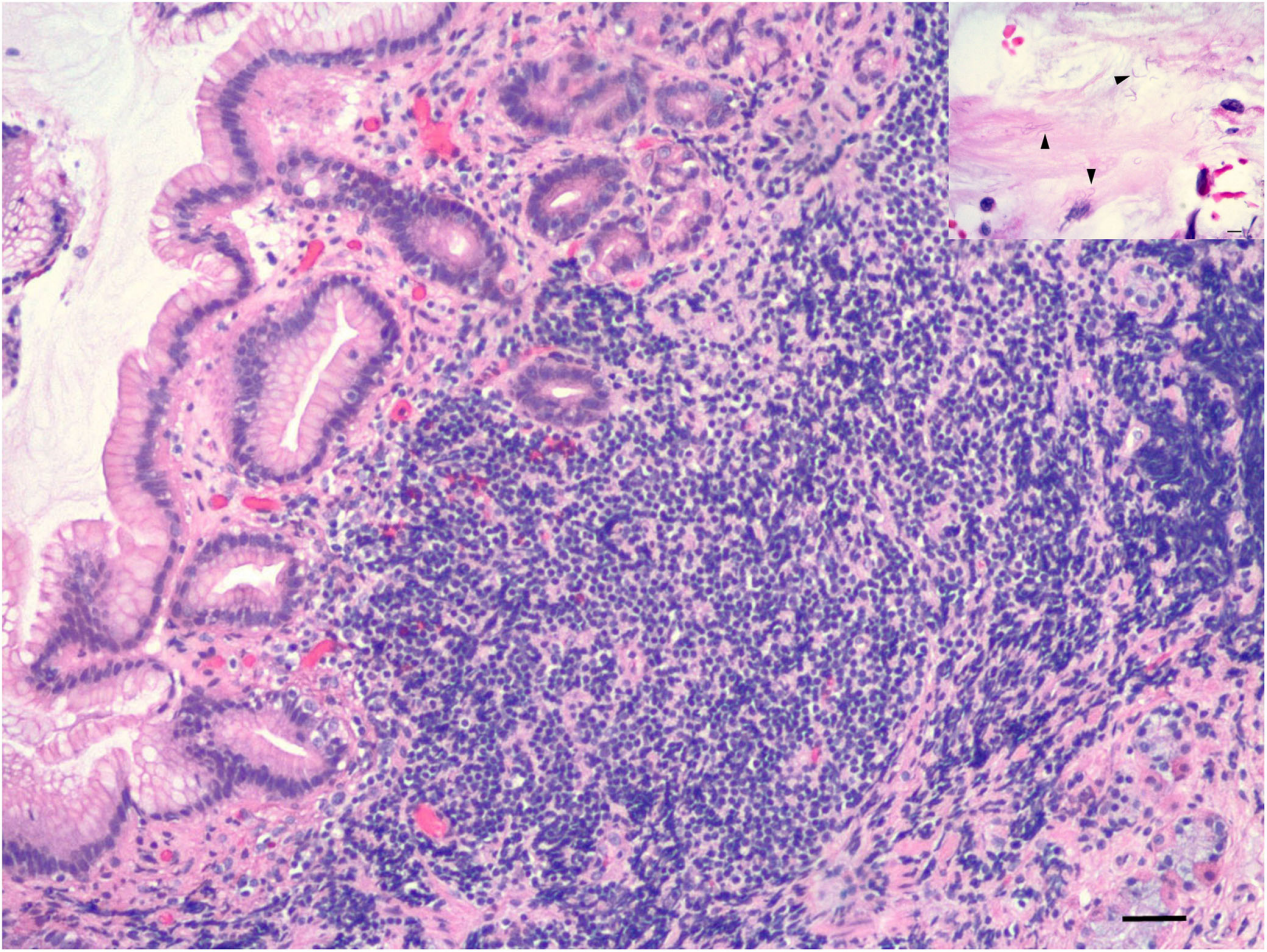
Histopathology of fundic cup biopsies. Stomach with lymphoplasmacytic gastritis, 10x magnification, scale bar: 100 μm. Inset shows spiral bacteria (*Helicobacter* spp.) (arrowheads) in surface mucus of stomach, 60x magnification, scale bar: 15 μm.

Endoscopy revealed several areas of thickening (1 cm) noted in the area of the fundus (Figure 2). These lesions grossly appeared to be involving the stomach wall and not intraluminal. Cup biopsy forceps were used to take multiple samples of the most prominent area of abnormality in two separate areas. Yellow-white fluid was noted from the biopsy sites in addition to blood. Espophageal endoscopy revealed normal structures. Histopathologic examination showed large lymphocytic aggregates in 3 out of the 4 samples. All four samples had a mild multifocal inflammation of lymphocytes, plasma cells, and eosinophils with few *Helicobacter*-like organisms (HLO) on the epithelial surface (Figure 3). Histologic diagnosis was lymphocytic-plasmacytic gastritis with *Helicobacter* spp. Further workup on isolating and specifying the *Helicobacter* species was not performed.

Based on the diagnostic findings, the patient was prescribed amoxicillin 400 mg BID for 21 days, clarithromycin 180 mg BID for 21 days, omeprazole 20 mg SID for 34 days, and Purina FortiFlora^®^, and Royal Canin^®^ GI diet.

**Figure 4:**
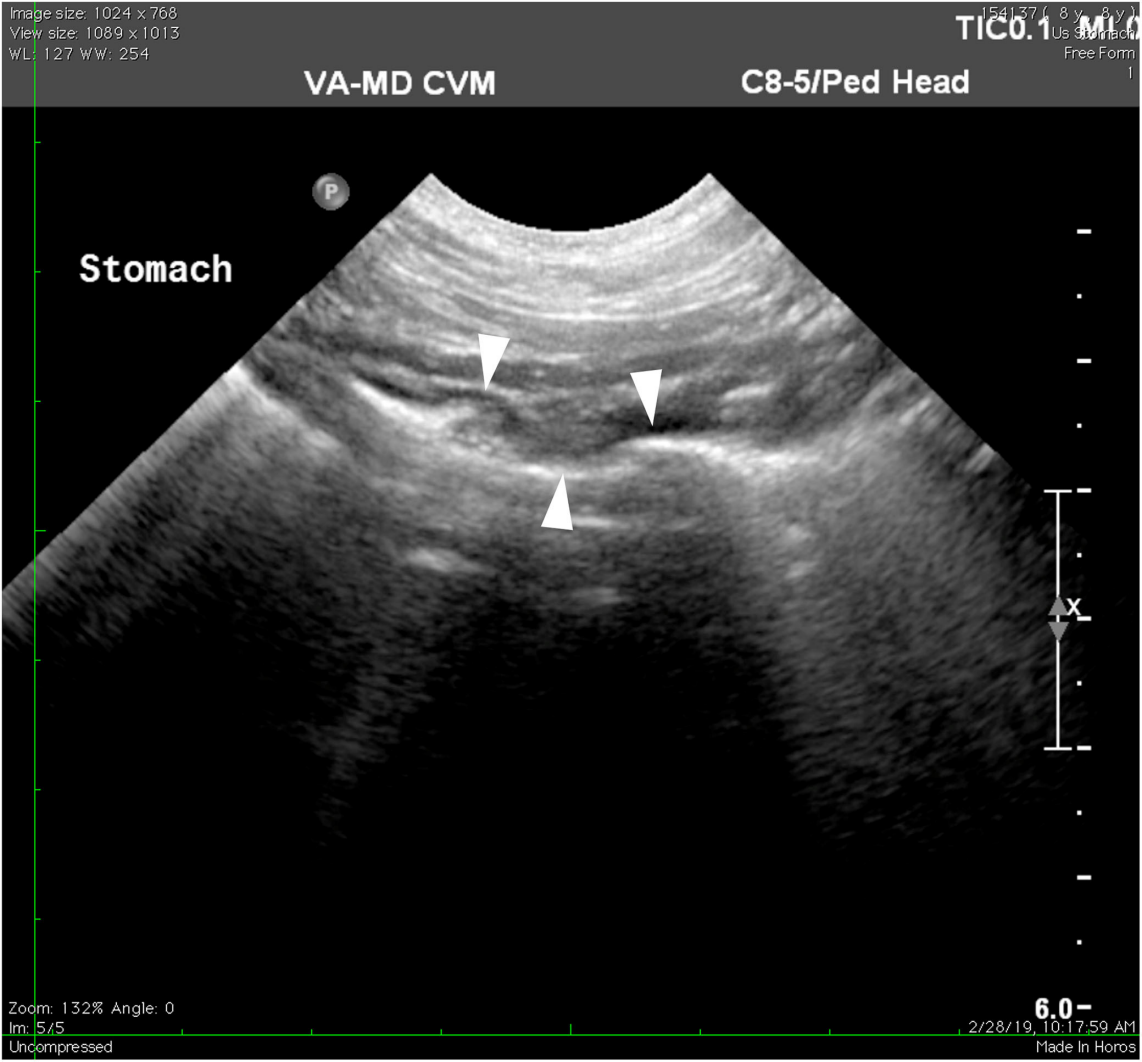
Ultrasonography of the stomach 27 days after starting antimicrobial therapy. Previously described fundic lesions appear to no longer be present in the stomach wall (arrowheads).

Nine days after the onset of medications, on follow-up telephone communications, the owner reported that the patient's vomiting had greatly decreased with only two instances of vomiting at home. Twenty-seven days after the onset of medications, the patient was re-evaluated at the community practice. The owner reported that the dog was doing very well at home with only one instance of vomiting. No abnormalities were found on the recheck physical examination. Transabdominal ultrasonography revealed that the previously described hypoechoic foci within the fundic lumen were no longer present, indicating that the gastric lesion was resolved (Figure 4).

The patient lived for another 2 years before dying of unrelated causes. At that time, the owner did not report any recurrence of the clinical signs.

## Discussion

In this case, the dog had multifocally raised mass-like nodules associated with *Helicobacter* spp. gastritis, which has not been previously documented in a canine. The mechanisms by which NHPHs were able to form raised lesions in this canine are unknown, but a potential theory that the authors have hypothesized would be through the formation of a biofilm. *Helicobacter* biofilm formation has been described *in vitro* and is suspected to play a role in human cases of chronic *Helicobacter pylori* gastritis (5). In human studies, *Helicobacter pylori* infection and growth consistent with biofilm formation have been found in gastric glands (5). Such a mechanism would account for the focal gross appearance of the current case. *Helicobacter* biofilms associated with chronic gastritis have been minimally described in veterinary species and warrant further investigation.

In human patients, nodular lesions associated with *Helicobacter pylori* have been described as a precursor to gastric neoplasia (6). However, progression to neoplasia can be avoided when timely *Helicobacter pylori* therapy is applied (6). In canines, *Helicobacter* spp. are often found in conjunction with gastric neoplasia (7). However, the infection has not been previously associated with nodular neoplastic precursors, and its role, if any, in the pathogenesis of gastric neoplasia in canines remains undiscovered (7, 8).

In this case, exact speciation of the NHPHs was not performed nor was a rapid urease test performed. *Helicobacter* species commonly isolated from canine gastric mucosa, include *H. felis, H. bizzozeronii, H. salomonis, H. bilis, H. heilmannii*, and *Flexispira rappini*, a closely related spirochete (3). Current knowledge of these species is that they behave similarly and cause similar lesions and clinical signs. Bacterial culture and PCR testing were not performed in this case as collecting the samples would have added another invasive procedure for this patient and was not deemed clinically necessary by the authors. *Helicobacter* spp. infections can be reliably diagnosed based on histologic appearance with the observation of the presence of the organism (3).

Abdominal ultrasonography was pursued early in this case as other non-invasive diagnostic tests failed to rule in the authors' differentials. The lesions found on ultrasound moved neoplasia higher up in the list of differentials based on the patient's signalment and lack of other findings. Endoscopy, with the intent to collect a biopsy sample for histopathology, followed suit, with the type of gross lesion in the present case being consistent with gastric adenocarcinoma, leiomyoma, leiomyosarcoma, and extramedullary plasmacytoma among others (8). Many of these differentials carry a poor-to-guarded long-term prognosis and may require surgical intervention (8). When the histopathology results did not support a neoplastic etiology, instead supporting NHPHs-associated lymphocytic-plasmacytic gastritis as the diagnosis, the authors pursued medical management as it was less invasive than performing another biopsy or surgical excision. Fortunately, the lesions showed resolution *via* ultrasonographical examination and clinical signs had resolved with the empirical treatment, so no further diagnostics nor interventions were clinically necessary. Even in the face of a gross nodular appearance, histopathology remains an important diagnostic tool for gastric lesions to determine the etiology and prognosis. In this case, histopathologic results showed lesions consistent with typical *Helicobacter* spp. gastritis and lacked findings associated with primary gastric neoplasia.

The potential limitation of this case is that endoscopy and endoscopic biopsy are not always readily available in the average general practice. Identifying the species of NHPHs involved may have bolstered the impact of this case for the betterment of understanding the role of *Helicobacter* spp. in canine gastritis; however, this was not proven clinically relevant in this patient.

There are still areas of discovery regarding NHPHs infections and canine gastritis. Based on the authors findings, *Helicobacter* spp. gastritis should remain on a differential list for canine patients experiencing chronic vomiting or those with mass-like gastric lesions on ultrasonographical or endoscopic examination and should not be ruled out by gross lesion appearance alone.

## Data availability statement

The original contributions presented in the study are included in the article/supplementary material, further inquiries can be directed to the corresponding author/s.

## Ethics statement

Ethical review and approval was not required for the animal study because of the animal was seen as a clinical patient at the Veterinary Teaching hospital and all documentation occurred within normal treatment/care for the patient. Written informed consent was obtained from the owners for the participation of their animals in this study.

## Author contributions

TG and CH contributed to writing the manuscript and literature review. MN contributed to the critical revision of the manuscript and managed the clinical case. TG and MN contributed to the final review. All authors contributed to the article and approved the submitted version.

## Funding

Open access publication fees provided by Virginia Tech University Libraries.

## Conflict of interest

The authors declare that the research was conducted in the absence of any commercial or financial relationships that could be construed as a potential conflict of interest.

## Publisher's note

All claims expressed in this article are solely those of the authors and do not necessarily represent those of their affiliated organizations, or those of the publisher, the editors and the reviewers. Any product that may be evaluated in this article, or claim that may be made by its manufacturer, is not guaranteed or endorsed by the publisher.
